# Resynthesizing *Brassica napus* with race specific resistance genes and race non-specific QTLs to multiple races of *Plasmodiophora brassicae*

**DOI:** 10.1038/s41598-024-64795-x

**Published:** 2024-06-25

**Authors:** Md. Masud Karim, Fengqun Yu

**Affiliations:** Saskatoon Research and Development Centre, Agriculture and Agri-Food Canada, 107 Science Place, Saskatoon, SK S7N 0X2 Canada

**Keywords:** Resynthesize, Clubroot resistance, Quantitative and qualitative resistance, Race specific, Race non-specific, *Brassica* spp., Plant breeding, Biotic

## Abstract

Clubroot disease in canola (*Brassica napus*) continues to spread across the Canadian prairies. Growing resistant cultivars is considered the most economical means of controlling the disease. However, sources of resistance to clubroot in *B. napus* are very limited. In this study, we conducted interspecific crosses using a *B. rapa* line (T19) carrying race-specific resistance genes and two *B. oleracea* lines, ECD11 and JL04, carrying race non-specific QTLs. Employing embryo rescue and conventional breeding methods, we successfully resynthesized a total of eight *B. napus* lines, with four derived from T19 × ECD11 and four from T19 × JL04. Additionally, four semi-resynthesized lines were developed through crosses with a canola line (DH16516). Testing for resistance to eight significant races of *Plasmodiophora brassicae* was conducted on seven resynthesized lines and four semi-resynthesized lines. All lines exhibited high resistance to the strains. Confirmation of the presence of clubroot resistance genes/QTLs was performed in the resynthesized lines using SNP markers linked to race-specific genes in T19 and race non-specific QTLs in ECD11. The developed *B. napus* germplasms containing clubroot resistance are highly valuable for the development of canola cultivars resistant to clubroot.

## Introduction

Canada is the world's leading canola (*Brassica napus*) producer, and canola remains the most profitable agricultural crop in Canada. However, this success has been threatened by the emerging clubroot disease caused by *Plasmodiophora brassicae* Woronin^1,2^. Addressing this disease in canola is of utmost priority to mitigate potential losses and achieve the industry's goal of increasing average yields to 52 bushels/acre. This goal is crucial to meet the global market demand of 26 million tons by 2025, as opposed to the current annual production of 20 million tons^3^.

Clubroot has continued to spread across the Canadian prairies over the last decade^4^, posing a serious long-term threat to canola production. The disease has been reported to cause about a 10–15% yield loss globally^5^. Based on the Williams differential system^6^, five pathotypes of *P. brassicae* (pathotypes 2, 3, 5, 6, and 8) were previously identified, with pathotype 3 being the most prevalent on canola in the prairie region^7^. The first clubroot-resistant canola cultivar in western Canada was released in 2009, followed by the release of the first generation of resistant cultivars from various breeding companies starting in 2010. These cultivars showed strong resistance to the old pathotypes of *P. brassicae* present in Canada. However, resistance in Canadian canola cultivars was soon overcome by new strains of *P. brassicae* identified in canola fields. Strains of *P. brassicae* collected in Canada have been classified into more than 30 pathotypes based on reactions on the Canadian Clubroot Differential (CCD)^2,8^. This rapid breakdown of resistance highlights the vulnerability of qualitative resistance controlled by major genes. To achieve more durable resistance, it is essential to develop new sources of resistance that combine both quantitative and qualitative resistance.

Genetic mapping of clubroot resistance (CR) genes has been extensively conducted in *Brassica* species to address resistance to strains of *P. brassicae* collected in canola fields in western Canada. Our group has identified major resistance genes (*Rcr1* to *Rcr10*) against both old and major new pathotypes such as 3A and 3D in *Brassica* species. We have developed robust SNP markers tightly linked to each of these resistance genes^9–17^. Recently, two major (*Rcr11* and *Rcr13*) and one minor QTL (*Rcr_C03-4*^*ECD10*^) were identified for resistance to 12 pathotypes (3A, 2B, 5C, 3D, 8E, 5G, 3H, 8J, 5L, 3O, 8P, and 5X) of *Plasmodiophora brassicae* in the *B. napus* cultivar ECD10 through genotyping-by-sequencing^18^. Identification of major genes conferring resistance to Canadian pathotypes in Brassica species has been also conducted at the University of Manitoba^19,20^ and the University of Alberta^21–27^. However, these major genes typically provide qualitative resistance, which is considered race-specific and can be rapidly overcome by the pathogen.

*B. napus*, originating from hybridization between *B. rapa* and *B. oleracea*, stands out as the most significant canola species globally, particularly in Canada. As mentioned earlier, sources of resistance in the A-genome derived from *B. rapa* have been extensively identified and are actively employed in the development of resistant canola cultivars in Western Canada. However, resistance sources in the C-genome from *B. oleracea* have not been widely utilized in canola breeding programs. This is primarily due to the challenge of transferring resistance directly from *B. oleracea* to *B. napus* through interspecific crosses, owing to reproductive barriers. Moreover, the availability of clubroot resistance sources in *B. oleracea* is limited. Genetic analysis of the CR genes in *B. oleracea* reveals that they are quantitative traits primarily controlled by quantitative trait loci (QTL)^28–30^, suggesting the potential for more durable, race non-specific clubroot resistance.

Two *B. oleracea* lines, ECD11 and JL04, demonstrated resistance to new strains of the clubroot pathogen during the screening of a large *B. oleracea* collection in our research group. The cabbage cultivar ECD11 (*B. oleracea* subsp. *capitata*, Badger Shipper) exhibited resistance to 15 out of 17 pathotypes in the CCD Set^8^ and other new pathotypes^2^, suggesting that ECD11 may possess genes with a broad spectrum of resistance. Recently, race non-specific QTLs were identified in ECD11^29^. Additionally, the kale breeding line JL04, originating from China, exhibited robust resistance to clubroot and likely carries race non-specific resistance genes/QTLs. In this study, we conducted the resynthesis of *B. napus* lines by crossing *B. oleracea* lines with a *B. rapa* line (T19) carrying three major resistance genes (*Rcr4*, *Rcr8* and *Rcr9*)^11^. Consequently, the resynthesized *B. napus* germplasms from this study harbor both quantitative and qualitative resistance genes.

The objectives of the current study were: (1) to resynthesize *B. napus* germplasms with qualitative resistance from A-genome species *B. rapa* and quantitative resistance from C-genome species *B. oleracea*, (2) to characterize the resynthesized germplasms for resistance to important races of *P. brassicae* identified in Western Canada, and (3) to confirm the presence of the genes/QTLs in the resynthesized germplasms using molecular markers.

## Results

### Development of the resynthesized and semi-resynthesized *B. napus* germplasms

**Figure 1:**
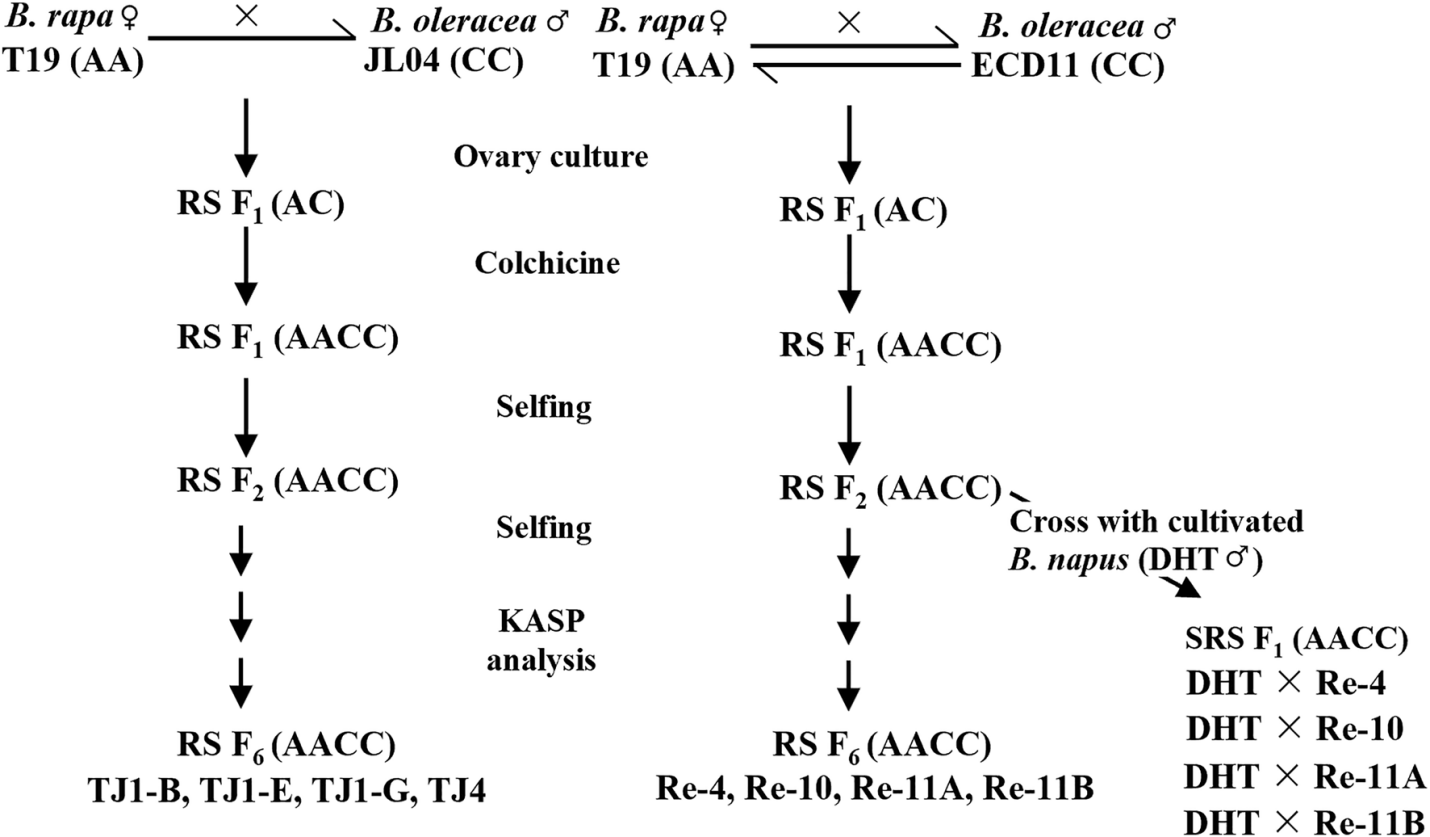
Schematic diagram showing the development process of clubroot resistant (CR) resynthesized (RS) and semi-resynthesized (SRS) *B. napus* (AACC). Reciprocal crosses were initiated between *B. rapa* (AA) line T19 and *B. oleracea* (CC) lines ECD11, and non-reciprocal crosses were made between *B. rapa* (AA) line T19 and *B. oleracea* (CC) lines JL04. Semi-resynthesized (SRS) *B. napus* (AACC) lines were created by crossing resynthesized (RS) F_2_ plants with doubled haploid (DH) *B. napus* canola line DH16516 (DHT).

In the cross set of T19 (AA) × ECD11 (CC), 137 ovaries were obtained from 221 pollinated flowers, resulting in an ovary setting rate of 61.9%. Thirteen F_1_ hybrid plants germinated from the transplanted ovaries on MS medium, and 10 plants survived after transplantation into the soil. Four F_1_ amphidiploid (AACC) plants (Re-4, Re-10, Re-11A, Re-11B) produced seeds following colchicine treatment (Fig. 1). In the reciprocal cross set of ECD11 × T19, 26 ovaries were obtained from 57 pollinated flowers, yielding an ovary setting rate of 45.6%. Three F_1_ hybrid plants germinated from the transplanted ovaries on MS medium, but only one plant survived after transplantation into the soil and could not produce seeds after colchicine treatment. Notably, a higher success silique set rate was observed using *B. rapa* as the female parent (61.9%) compared to *B. oleracea* (45.6%). Therefore, crosses with *B. rapa* as the female were exclusively employed in the T19 and JL04 crosses, utilizing 10 plants of T19 and 6 plants of JL04.

The cross set for T19 (AA) × JL04 (CC) resulted in 230 ovaries from 269 pollinated flowers, achieving an ovary setting rate of 85.5%. Thirty-five hybrid plants germinated from the transplanted ovaries on MS medium, 34 plants survived after transplantation into the soil, and 4 F_1_ amphidiploid (AACC) plants (TJ1-B, TJ1-E, TJ1-G, TJ4) produced seeds after colchicine treatment (Fig. 1).

In total, 51 F_1_ hybrid plants germinated, with 45 surviving in the soil, and ultimately, 8 hybrid plants successfully produced seeds from a total of 547 pollinations across the three cross combinations (Table 1). The cross ability percentages for these three combinations (T19 × ECD11, ECD11 × T19, T19 × JL04) were 1.8%, 0.0%, and 1.5%, respectively.

**Table 1:** Cross combination and cross ability of interspecific hybridization between *Brassica rapa* (AA) parental line T19 and *Brassica oleracea* (CC) parental lines ECD11 and JL04.

Cross combination (♀ × ♂)	No. of parental line used	Flowers pollinated (a)	Ovary set (rate, %)	F_1_ (AC) germinated	F_1_ (AC) survived in soil	F_1_-hybrid (AACC) produced seeds (b)	Cross ability (b/a, %)
T19 × ECD11	T19: 6 plants ECD11: 3 plants	221	137 (61.9)	13	10	4	1.8
ECD11 × T19	T19: 6 plants ECD11: 3 plants	57	26 (45.6)	3	1	0	0.0
T19 × JL04	T19: 10 plants JL04: 6 plants	269	230 (85.5)	35	34	4	1.5
Total		547	393 (78.1)	51	45	8	1.5

a = number of flowers pollinated in each cross combination, b = number of hybrid plants successfully produced in each cross combination, b/a × 100% = cross ability of each cross combination in percentage.

### Seed fertility of the developed resynthesized and semi-resynthesized plants

**Figure 2:**
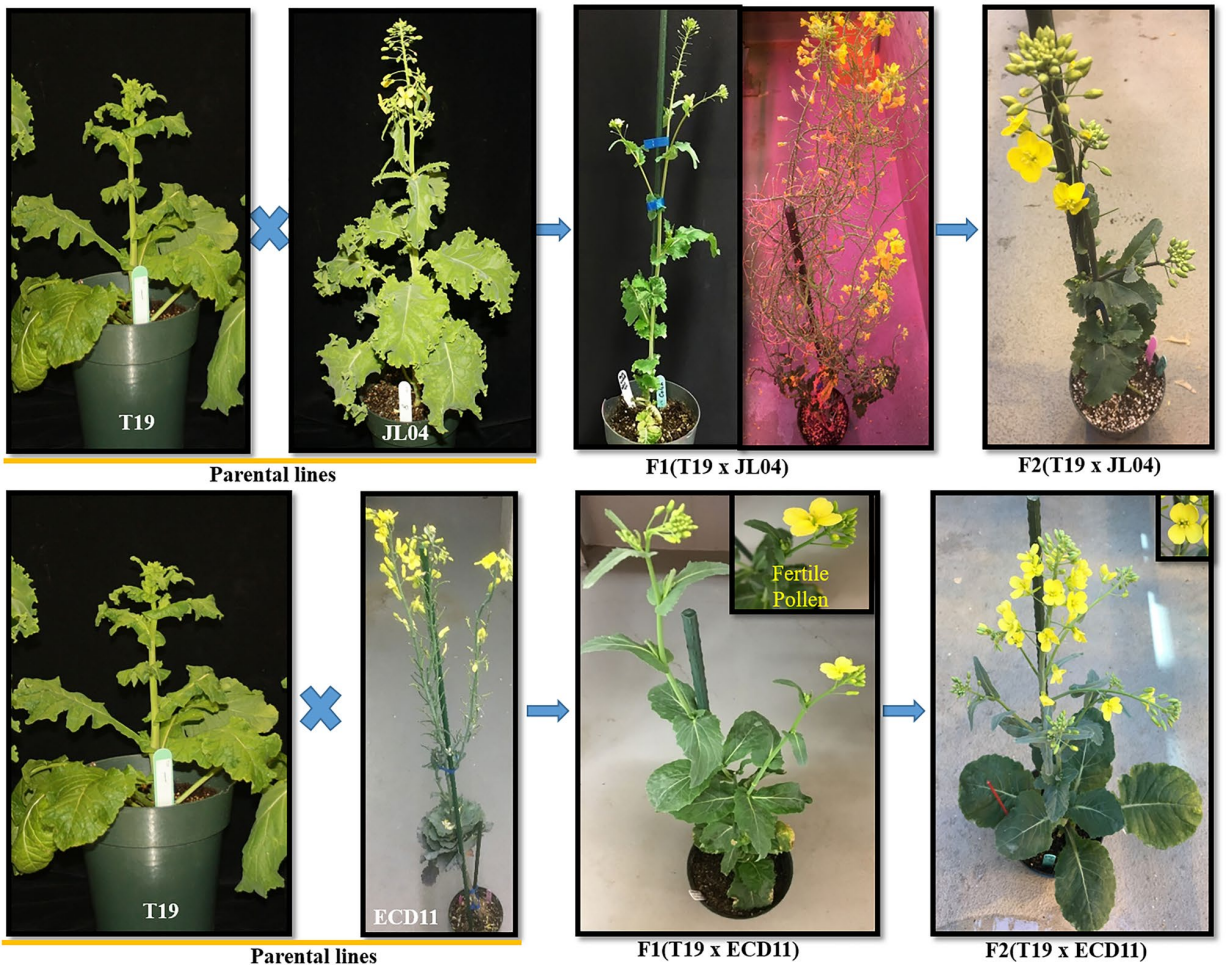
Resynthesized *Brassica napus* (AACC) generations derived from interspecific hybridization between *Brassica rapa* (AA) parental line T19 and *Brassica oleracea* (CC) parental lines ECD11 and JL04. To double the chromosome numbers and restore pollen fertility in the resynthesized F_1_ (AC), colchicine treatment was applied to the roots/leaf axils of hybrid plants. Amphidiploids (AACC) were identified by the presence of fertile pollen and the development of fully formed stamens.

Hybridity of the F_1_ plants was confirmed through careful assessment of morphological characteristics such as vigorous growth, plant height, leaf size and shapes, flower size, and pollen viability. Eight F_1_ hybrid plants successfully produced fertile flowers after chromosome doubling with colchicine treatment. Upon self-pollination, F_2_ seeds were successfully generated, designating these plants as amphidiploid (AACC) *B. napus* (Fig. 2). To ensure the stability of the newly synthesized lines, selection of self-compatible plants and selfing were continued up to the F_6_ generation. Approximately 10 plants per line were grown from the T19 × ECD11 cross. Re-10, Re-11A and Re-11B exhibited higher seed set in advanced generations, while the Re-4 line consistently showed poor seed set. Similarly, among the four lines from the T19 × JL04 cross combination, TJ1-B, TJ1-E and TJ1-G demonstrated higher seed set up to the F_4_ generations (Table 2), while TJ4 exhibited poor seed set. Although resynthesized plants showed poor seed fertility until advanced generations, all semi-resynthesized plants exhibited normal seed fertility in their early generation, crossing with DH16516 (Fig. 1, Table 2).

**Table 2:** Amount of seed (g) produced from resynthesized and semi-resynthesized *Brassica napus* (AACC) lines of different generations.

Plant ID	Cross combination (♀ × ♂)	Amount of seed (g)					
		F_1_	F_2_	F_3_	F_4_	F_5_	F_6_
Re-4	T19 × ECD11	Ovary culture	0.07	10 seed	0.06	No seed	No seed
Re-10	T19 × ECD11	Ovary culture	0.49	1.03	5 seed	0.41	0.07
Re-11A	T19 × ECD11	Ovary culture	7 seed	2.09	2.68	1.71	0.18
Re-11B	T19 × ECD11	Ovary culture	11 seed	1.92	0.89	4.00	1.33
TJ1-B	T19 × JL04	Ovary culture	0.09	0.51	1.00	–	–
TJ1-E	T19 × JL04	Ovary culture	0.09	0.29	0.68	–	–
TJ1-G	T19 × JL04	Ovary culture	0.09	1.05	9.08	–	–
TJ4	T19 × JL04	Ovary culture	0.01	–	–	–	–
Semi-resynthesis	DH16516 × Re-4(F_2_)	3.9 g	–	–	–	–	–
	DH16516 × Re-10(F_2_)	5.0 g	–	–	–	–	–
	DH16516 × Re-11A(F_2_)	2.5 g	–	–	–	–	–
	DH16516 × Re-11B(F_2_)	4.9 g	–	–	–	–	–

### Characterization of resynthesized and semi-resynthesized *B. napus* lines with *P. brassicae* identified in Western Canada

Seven resynthesized and four semi-resynthesized F_1_ lines, which produced a substantial amount of seeds (Table 2), parental lines, and susceptible control (DH16516 and NRC11-24) were selected for inoculation with selected races of *P. brassicae*, as shown in Table S1.

**Figure 3:**
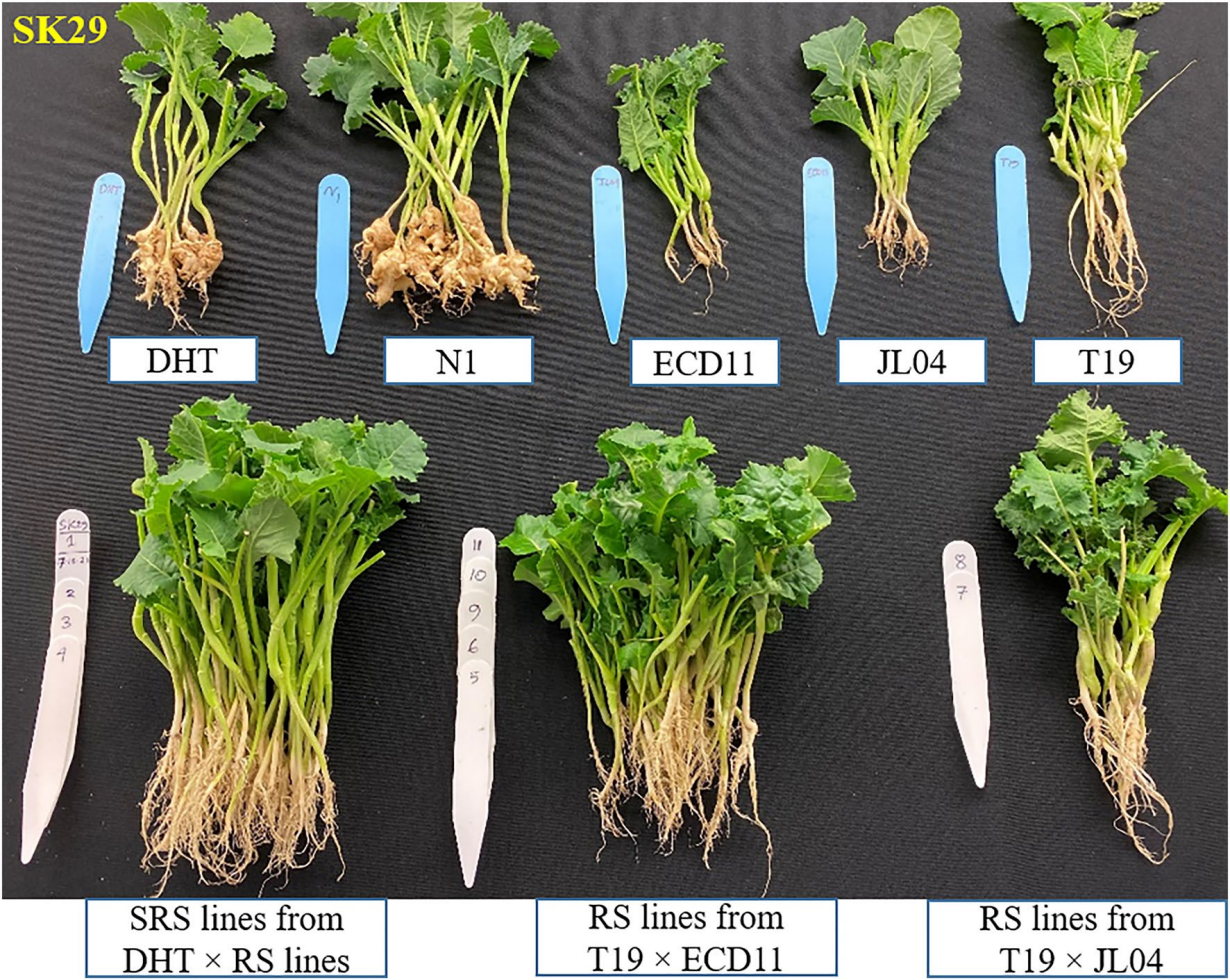
An evaluation of resynthesized (RS) *Brassica napus* (AACC) lines derived from two crosses, T19 × ECD11 and T19 × JL04, and semi-resynthesized (SRS) lines derived from DH16516 (DHT) × (T19 × ECD11) was conducted for clubroot resistance against 8 strains of *Plasmodiophora brassicae*. The root phenotypes of resynthesized lines, semi-resynthesized lines, parental lines (T19, ECD11, JL04) and susceptible (S) control lines DH16516 and NRC11-24 (N1) after six weeks of inoculation with strain SK29.

**Figure 4:**
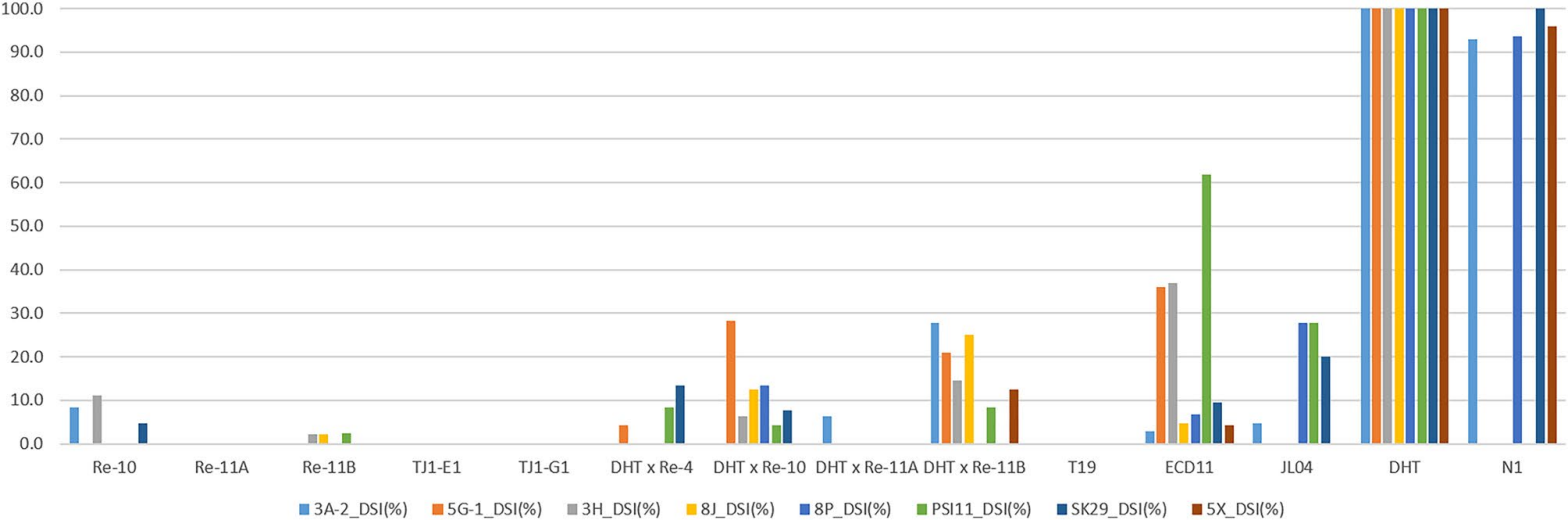
Evaluation of resynthesized *Brassica napus* (AACC) lines of different generations; Re-10, Re-11A and Re-11B lines were derived from the cross T19 × ECD11; lines TJ1-B1 and TJ1-G1 were derived from the cross T19 × JL04; and four semi-resynthesized lines derived from DH16516 (DHT) × (T19 × ECD11) for clubroot resistance. Distribution of disease severity indexes (DSIs) of resynthesized, semi-resynthesized, parental (T19, ECD11, JL04) and susceptible (S) control lines DH16516 and NRC11-24 (N1) against 8 strains: 3A, 5G, 3H, 8J, 8P, PSI11, SK29 and 5X, representing 8 races of *Plasmodiophora brassicae*.

A total of 1,060 resynthesized and semi-resynthesized plants, along with parental lines and control plants, were tested against strains representing eight races of *P. brassicae* identified in Western Canada. All resynthesized lines exhibited high resistance against all eight strains/pathotypes (P.41-14/3H, 4-15/3A, CDCS/5G, LG02/5X, F.12-15/8J, Leduc C#37/8P, SK29, PSI11) (Figs. 3 and 4, Table S2): 3A (%DSI = 0.0–8.3), 5G (%DSI = 0.0), 3H (%DSI = 0.0–11.1), 8J (%DSI = 0.0–4.2), 8P (%DSI = 0.0), PSI11 (%DSI = 0.0–4.2), SK29 (%DSI = 0.0–4.8), and 5X (%DSI = 0.0). Similarly, semi-resynthesized lines also demonstrated resistance against all tested strains (Figs. 3 and 4, Table S2): 3A (%DSI = 0.0–27.8), 5G (%DSI = 4.2–28.2), 3H (%DSI = 0.0–14.6), 8J (%DSI = 0.0–25.0), 8P (%DSI = 0.0–13.3), PSI11 (%DSI = 0.0–8.3), SK29 (%DSI = 0.0–13.3), and 5X (%DSI = 0.0–12.5).

The A-genome parent (T19) exhibited a high level of resistance against all eight strains (%DSI = 0.0), while resistance varied in C-genome parents ECD11 and JL04. ECD11 showed high resistance against 3A (%DSI = 2.8), 8J (%DSI = 4.8), 8P (%DSI = 6.7), SK29 (%DSI = 9.5), 5X (%DSI = 4.2), and moderate resistance against 5G (%DSI = 36.1), 3H (%DSI = 37.0), and susceptibility against PSI11 (%DSI = 61.9). JL04 showed high resistance against 3A (%DSI = 4.8), 5G, 3H, 8J, 5X (%DSI = 0.0), partial resistance against 8P (%DSI = 27.8), PSI11 (%DSI = 27.8), and SK29 (%DSI = 20.0).

DH16516 was highly susceptible against all eight strains (%DSI = 100), whereas NRC11-24 showed high resistance to 3H, 5G, 8J, PSI11 (%DSI = 0) and susceptibility against 3A (%DSI = 92.9), 5X (%DSI = 95.8), 8P (%DSI = 93.8), SK29 (%DSI = 100) (Fig. 4).

### Confirmation of the presence of clubroot resistance genes/QTLs in the newly resynthesized *B. napus* lines

A total of 21 resynthesized plants (3 plants/line) from 7 resynthesized lines (Re-4, Re-10, Re-11A, Re-11B, TJ1-B, TJ1-E, TJ1-G) were selected to confirm the presence of identified clubroot resistance genes and QTLs: *Rcr4*, *Rcr8*, *Rcr9* from A-genome and *Rcr_C03-1*, *Rcr_C08-1* from C-genome (Table 3, Table S3). SNP markers associated with the resistance alleles were tested in the resynthesized lines from both crosses, T19 × ECD11 and T19 × JL04 (Table S3, Fig. S2). The results confirmed the presence of the resistance genes and QTLs in the resynthesized *B. napus* lines, also showing that JL04 may carry *Rcr_C03-1* and *Rcr_C08-1*.

**Table 3:** SNP markers linked with 5 clubroot resistance genes/QTLs of A- and C-genome were used for marker confirmation in resynthesized *Brassica napus* (AACC) lines along with A- and C-genome susceptible control.

Chr	QTL/gene name	Marker name	Position	KASP results	
				Resyn. lines from T19 × ECD11	Resyn. lines from T19 × JL04
A03	***Rcr4***	A3-12	V1.5_24375572	*Rcr4*	*Rcr4*
A02	***Rcr8***	A2-Y3	V1.5_18080035	*Rcr8*	*Rcr8*
		A2-Y6	V1.5_18504402	*Rcr8*	*Rcr8*
		DTS-12	DM_19953009	*Rcr8*	*Rcr8*
		DTS-25	DM_22090678	*Rcr8*	*Rcr8*
A08	***Rcr9***	A8-Y07	V1.5_9015777	*Rcr9*	*Rcr9*
		M22	V1.5_10659607	*Rcr9*	*Rcr9*
		M23	V1.5_10705386	*Rcr9*	*Rcr9*
C03	***Rcr_C03-1***	DC3-19	D134_11174237	*Rcr_C03-1*	*Rcr_C03-1*
		DC3-20	D134_11339341	*Rcr_C03-1*	*Rcr_C03-1*
		DC3-22	D134_12650068	*Rcr_C03-1*	*Rcr_C03-1*
C08	***Rcr_C08-1***	DC8-5	D134_23698705	*Rcr_C08-1*	*Rcr_C08-1*
		DC8-7	D134_24249890	*Rcr_C08-1*	*Rcr_C08-1*

A-genome markers were designed based on the reference genome of *B. rapa* (sequence_v1.5.fa)^31^ downloaded from http://brassicadb.org/brad/downloadOverview.php, and the *B. napus* reference genome 'Darmor-bzh-v4.1’^32^ was obtained from https://www.genoscope.cns.fr/brassicanapus/data/. For C-genome markers, the reference genome of the DH line D134 (cabbage; *B. oleracea* var. *capitata*)^33^ was utilized and downloaded from https://db.cngb.org/search/?q=CNP0000469.

## Discussion

Hybrid embryos encounter growth restrictions due to the absence of appropriate endosperm^34–37^. To overcome this limitation, techniques such as embryo, ovule, and ovary culture are commonly employed for embryo rescue in interspecific hybridization of *Brassica* species. Ovary culture proves effective when *B. rapa* is used as the female parent in crosses like *B. rapa* (♀) × *B. oleracea* (♂). However, it falls short in the reciprocal cross of *B. oleracea* (♀) × *B. rapa* (♂)^37–39^. In our study, we observed that *B. rapa* line T19 exhibited higher cross ability when used as a female parent, aligning with findings from previous research. Notably, there have been exceptions to this pattern, as reported in instances where resynthesized *B. napus* was successfully produced in both crossing directions using a *B. oleracea* line, CRGC3-1, as a parent^40,41^.

Resynthesized lines serve as valuable genetic resources in rapeseed breeding, contributing genetic diversity and crucial traits such as disease resistance (clubroot disease, sclerotinia stem rot, verticillium wilt, blackleg disease), yield, heterosis, insect resistance, pod shatter resistance, early flowering, and drought tolerance^42^. While some artificial rapeseeds were initially used solely as breeding materials due to low yields^36,43,44^, improved pollen and seed fertility have been achieved in advanced generations^41,45,46^. Consequently, selection lines from advanced generations with enhanced self-compatibility and seed fertility can be directly utilized for varietal development.

Resynthesized rapeseed lines exhibit self-incompatibility in the early generations, since they inherit functional S-alleles from both parent species, resulting in self-incompatibility in the offspring generations. The breakdown of self-incompatibility can occur due to not only loss-of-function mutations of alleles at the S-locus but also genetic interactions between specific S-alleles and unlinked modifiers^47^. In this study, although both parents of A and C genome used to produce the resynthesized lines were self-incompatible, self-compatible plants were selected during subsequent generations' selection processes and the self-compatibility trait improved in the advanced generations of the resynthesized lines. In semi-resynthesized F_1_ generation, it is expected that the S-alleles become heterozygous with non-functional and functional alleles provided by the respective parent, and there will be segregation of self-incompatible and self-compatible individuals in the F_2_ and subsequent generations of semi-resynthesized rapeseed lines. Unexpectedly, however, all our F_1_ semi-resynthesized rapeseed lines showed self-compatibility. Selection of the F_2_ and subsequent generations of semi-resynthesized rapeseed lines is underway; there is a need to select not only clubroot-resistant, but also self-compatible plants in the selection process as self-compatibility is an important breeding goal in rapeseed breeding.

In this study, earlier generations of resynthesized lines exhibited poor seed set, consistent with previous findings^41,48,49^. However, wide variation in seed set was observed in advanced generations, presenting opportunities for incorporation into canola breeding programs. Meiotic irregularities affecting genome stability contribute to low fertility in resynthesized *B. napus*^50^. These challenges can be addressed by selecting lines with improved seed fertility and by developing semi-resynthesized lines^41^. Consequently, four semi-resynthesized *B. napus* lines were developed by crossing resynthesized lines (Re-4, Re-10, Re-11A, and Re-11B) with a DH *B. napus* canola line, DH16516. While advanced generations of three resynthesized lines (Re-10, Re-11A, and Re-11B) showed good seed fertility, Re-4 did not. On the other hand, semi-resynthesized *B. napus* lines derived from all four resynthesized lines exhibited good seed fertility in their early generations. Thus, both selected resynthesized and all semi-resynthesized lines hold promise for integration into canola breeding programs.

The fertility of resynthesized lines is genotype-dependent, and due to frequent sterility in early generations, not all resynthesized lines may survive to advanced self-pollinated generations^42^. Notably, two resynthesized lines, Re-4 and TJ4, did not yield satisfactory self-pollinated seeds compared to the other six lines (Re-10, Re-11A, Re-11B, TJ1-B, TJ1-E, TJ1G). This variability in seed fertility not only exists among different lines but also among different plants of the same line, aligning with findings from previous studies^41^. Early generations of resynthesized *B. napus* often involve aneuploidy and gross chromosomal rearrangements. Notably, seed yield and pollen viability show an inverse correlation with increasing aneuploidy^51^.

In this study, our aim was to combine qualitative and quantitative resistance from *B. rapa* and *B. oleracea* into the *B. napus* resynthesized lines, resulting in lines that demonstrated a broad range of resistance to multiple races of *P. brassicae*. A previous study identified variation in quantitative traits, such as flowering time, attributed to structural rearrangements of chromosomes in resynthesized *B. napus*^52^. Interestingly, the stability of qualitative traits like self-incompatibility^53^ and clubroot resistance^54^ has often been observed in the self-pollinated progeny of resynthesized *B. napus* plants. While a loss of genomic regions carrying clubroot (CR) resistance (6–13%) has been reported during the development of self-pollination (S0 to S1 families) in resynthesized lines due to meiotic anomalies^49^, our current study did not observe any CR resistance loss in all homozygous resynthesized lines, as they maintained high resistance against all eight strains. Furthermore, four F_1_ semi-resynthesized lines were developed by crossing resynthesized lines with the clubroot-susceptible DH line DH16516. The resynthesized lines showed higher resistance (DSI = 0–11.1%) compared to the F_1_ semi-resynthesized *B. napus* lines (DSI = 0.0–28.2%). We observed some susceptible plants in the F_1_ semi-resynthesized *B. napus* lines; the reason for this is yet to be determined.

Meiotic anomalies and homoeologous pairing of chromosomes in the early generations of resynthesized Brassica allopolyploids can lead to structural rearrangements with the loss or gain of chromosomes^50,51,55–57^. An example is the European winter clubroot resistance (CR) canola cultivar ‘Mendel’, developed from a resynthesized *B. napus* line crossed from *B. oleracea* ECD15 × *B. rapa* ECD04^54^. ‘Mendel’ was initially expected to possess three dominant CR genes inherited from its diploid parent ECD04^58–60^. However, genetic mapping revealed that 'Mendel' had two dominant CR genes^61^, suggesting a potential loss of the other gene during the breeding process. Similar loss of CR genes has been reported in the breeding of a rutabaga line^62^. On the other hand, two *B. rapa* CR loci (*Crr1* and *Crr2*) were efficiently transferred through the development of resynthesized *B. napus* lines and properly introduced into the recurrent parental lines using CR loci-linked markers^63^. There were 6, 4 and 2 genes encoding TIR-NBS-LRR class disease resistance proteins in the *Rcr4*, *Rcr8*, and *Rcr9* flanking regions in T19, and developed SNP markers linked to these QTLs^11^. Recently, we first confirmed that clubroot resistance genes *Rcr1*, *Rcr2*, *Rcr4*, and *CRa* from *B. rapa* vegetables and the resistance gene from *B. napus* oilseed rape cv. “Mendel” on chromosome A03 were identical in their coding regions after cloning and functional verification of the clubroot resistance gene *Rcr1*^64^. In our recent study, two race no-specific QTLs (*Rcr_C03-1*, *Rcr_C08-1*) were identified in ECD11^29^. There were 10 and 4 genes encoding TIR-NBS-LRR and CC-NBS-LRR class disease resistance proteins in the *Rcr_C03-1* and *Rcr_C08-1* flanking regions, and developed SNP markers linked to these QTLs. All targeted loci, including *Rcr4*, *Rcr8*, and *Rcr9* in the resynthesized *B. napus* lines, were confirmed using SNP markers closely linked to the genes. C-genome CR QTLs (*Rcr_C03-1*, *Rcr_C08-1*) from ECD11 were also confirmed in the resynthesized lines using tightly linked SNP markers. However, no studies on genetic mapping for the identification of QTLs in JL04 have been carried out so far. Interestingly, some of the linked SNP markers for the QTLs in ECD11 were identified in the resynthesized lines from T19 × JL04. Further research on genetic mapping is needed to confirm these results and identify novel QTLs in JL04.

## Conclusion

In this current study, we successfully developed eight resynthesized *B. napus* lines from T19 × ECD11 and JL04, achieving a broad spectrum of resistance from both qualitative and quantitative resistance backgrounds. All targeted loci were confirmed in the resynthesized lines using tightly linked markers. The germplasms and information obtained from this project are now available to canola breeders for rapid incorporation into their canola variety development programs (https://agriculture.canada.ca/en/science/technology-transfer-and-licensing-agriculture/evaluation-canola-breeding-lines-clubroot-resistance).

## Materials and methods

### Plant materials

A CR-resistant self-incompatible *B. rapa* breeding line, T19, derived from the German turnip cultivar ‘Pluto’, was developed at the Saskatoon Research and Development Centre, Agriculture and Agri-Food Canada (SRDC, AAFC), in Saskatoon, Saskatchewan, Canada. The seeds of the resistant *B. rapa* line (T19), crossed with two self-incompatible *B. oleracea* lines, ECD11 and JL04, were provided by Dr. G. R. Dixon (The University of Warwick, Wellesbourne, Warwick, UK), and Dr. Zhen Huang (Northwest A&F University, Yangling, Shaanxi, China), respectively.

### Reciprocal crosses, embryo rescue and chromosome doubling

Seedlings of the parental *B. rapa* (T19) and *B. oleracea* (ECD11, JL04) were grown in a greenhouse at the SRDC, AAFC. Reciprocal crosses were initiated between *B. rapa* (AA) line T19 and *B. oleracea* (CC) lines ECD11 and JL04. The parental lines T19, ECD11, and JL04 were grown in the greenhouse for two weeks and then exposed to a 3-month vernalization period at a cold room (+ 4 °C) to synchronize flowering time. Reciprocal crosses were conducted using 6 plants of T19 × 3 plants of ECD11 and 10 plants of T19 × 6 plants of JL04. Emasculation was carried out on floral buds 1–2 days before anthesis. The emasculated buds were promptly dusted with fresh pollen grains collected from the male parents (*B. oleracea* ECD11 and JL04). Pollinated flowers were isolated in thin paper bags, and ovaries bearing ovules were collected at 16–20 days after pollination (DAP) for F_1_ embryo rescue.

The embryo rescue technique, adapted from the method described by Inomata^38^ with some modifications, involved the sterilization of ovary surfaces. Ovaries were sterilized with 70% ethanol for 3 min, followed by treatment with a 10% solution of sodium hypochlorite for 10 min and rinsed twice with sterile distilled water. The sterilized ovaries were placed on MS medium^65^, aseptically supplemented with 1% sucrose and 0.8% agar, adjusted to pH 5.8. Plastic petri dishes (9.0 cm × 1.5 cm) were used for the cultures, which were then placed in a growth chamber maintained at 22 °C with a 16-h photoperiod (7:00 am to 11:00 pm). After 20–30 days, regenerating embryos were transferred to basal MS agar medium in a plant tissue culture container (10.0 cm × 7.5 cm × 7.5 cm) for root and shoot development. Once the regenerated plantlets reached a height of 3–4 inches, they were removed from culture and transplanted into four-inch pots containing Osmocote potting mix (Everris NA Inc.; Dublin, OH, USA) and covered with a transparent plastic cap. The plants were then placed in a growth chamber maintained at 22 °C with a 12-h photoperiod for two weeks before being transferred into six-inch pots containing Sunshine #3 soilless mix (Sun Gro Horticulture Canada Ltd.; Seba Beach, AB) (Fig. S1).

To restore seed fertility in the F_1_ hybrids, the roots of each F_1_ plant were submerged in a 3.4 g/L solution of colchicine for 1.5 h, then thoroughly washed with water and transplanted into the soil to double the chromosome numbers, following a previously described method^66^. In cases where F_1_ plants failed to produce fertile pollen, a 0.05% colchicine solution was applied to the leaf axils of hybrid plants, as described by Chen et al.^34^. Amphidiploids (AACC) were identified by the presence of fertile pollen and the development of fully formed stamens. Seed fertility (seeds/pod) in the early generations of the resynthesized *B. napus* lines was low due to self-incompatibility. To obtain self-pollinated seeds, plants with open flowers and unopened buds were enclosed in an airtight plastic bag, which was then filled with CO_2_ to raise the internal concentration to optimize the conditions for flower development and pollen production, which can ultimately lead to improved seed production. After 3–4 h, the bag was removed and replaced with a pollinating bag. Seeds were increased for further experiments. The resynthesized F_1_ plants were self-pollinated to produce subsequent progenies, from F_2_ to F_6_. As it was challenging to produce self-pollinated seeds in some resynthesized lines from T19 × ECD11; selected F_2_ plants were crossed with the doubled haploid (DH) *B. napus* canola line DH16516, developed by Dr. Séguin-Swartz at the SRDC, AAFC, to obtain semi-resynthesized F_1_ progeny (Fig. 1).

### Evaluation of newly resynthesized and semi-resynthesized *B. napus* lines for clubroot resistance

Strains of *P. brassicae* collected from canola fields in Alberta, Saskatchewan, and Manitoba were provided by Dr. S. E. Strelkov at the University of Alberta, Drs. A. Akhavan and B. Ziesman at the Government of Saskatchewan, and Dr. L. A. Murphy and Mr. X. Guo at Pest Surveillance Initiative, Canada (Table S1). The strains were characterized with five *B. napus* lines (unpublished data) carrying single clubroot resistance genes *Rcr1*, *Rcr3*, *Rcr8*, *Rcr9* and *Rcr10*, respectively^10,11,16,17^. These strains represent eight races of *P. brassicae* in the canola fields in Western Canada (Table S1). In addition, the strains from Alberta were characterized with the Canadian Clubroot Differentials^8^.

The newly resynthesized and semi-resynthesized *B. napus*, the parental lines, and control plants were tested for clubroot resistance with the *P. brassicae* strains in a greenhouse at the SRDC, AAFC. The inoculum preparation method used in this study was as described by Karim and Yu^29^. Seedlings of the susceptible canola line DH16516 or *B. napus* line NRC11-24 (Nutrien Ag. Solutions, Saskatoon, Saskatchewan) with a known resistance gene *Rcr1* were inoculated and maintained under controlled conditions to increase clubroot galls. Clubbed roots were harvested from infected plants after 5–6 weeks and stored at −20 °C. Fresh inoculum of each pathotype was prepared by softening about 5 g of frozen club in distilled water for 1–2 h, homogenizing it in a blender for 2 min, and straining it through 2–3 layers of nylon mesh cloth. The resulting spore suspension was diluted with deionized water to achieve a final concentration of 1 × 10^7^ resting spores/mL.

The inoculation method used in this study was as described by Karim and Yu^29^. Seeds of the resynthesized, semi-resynthesized lines, their parental lines T19, ECD11, and JL04, and the two controls DH16516 and NRC11-24 were sown into Sunshine #3 soilless mix (Sun Gro Horticulture Canada Ltd.; Seba Beach, AB) with Osmocote (Everris NA Inc.; Dublin, OH, USA) in 32-pot inserts held by trays (The HC Companies; Twinsburg, OH, USA). Adequate water was added to each tray to soak the soilless mix overnight. Seven days after planting, inoculation was performed by adding 15 ml of inoculum (1 × 10^7^ spores/ml) into each pot with 6–9 seedlings of each line with two replications. The inoculated plants were grown in a growth chamber set at 22/18 °C day/night temperature with a 16-h photoperiod. The pots were kept moist by adding water whenever necessary. Six weeks after inoculation, plant roots were examined for clubroot symptoms. Clubroot severity was evaluated on a 0 to 3 scale^67^, where 0 = no clubbing, 1 = a few small clubs (small galls on fewer than one-third of the roots), 2 = moderate clubbing (small to medium-sized galls on between one-third and two-thirds of the roots), and 3 = severe clubbing (medium to large-sized galls on over two-thirds of the roots). A disease severity index (DSI) was calculated for each line using the method of Horiuchi and Hori^68^:

$$\text{DSI}=\frac{\sum (\text{rating class}) \times (\#\text{ plants in rating class}) }{\text{total }\#\text{ plants in treatment }\times 3}\times 100$$

### Kompetitive allele specific PCR (KASP) analysis

Young leaves from resynthesized *B. napus* lines, parental lines, and A- and C-genome susceptible control lines were harvested for DNA extraction. The leaves were freeze-dried in a Freezone 6 dryer (Labconco Corp, Kansas City, MO) for 48 h and ground using the Mixture Mills 300 (Retsch Inc., Newtown, PA). DNA extraction was carried out with the DNeasy 96 Plant Kit (Qiagen, Toronto, ON, Canada) following the DNeasy plant handbook from QIAGEN. The extracted DNA was quantified using a NanoVue Plus spectrophotometer (GE Healthcare, Piscataway, NJ), diluted to 10 ng/μl, and stored at −20 °C until subsequent use for genotyping.

The *B. rapa* parental line T19 carried race-specific resistance genes *Rcr4*, *Rcr8*, and *Rcr9*^11^. Two QTLs, *Rcr_C03-1* and *Rcr_C08-1*, conferring race non-specific resistance to *P. brassicae* races in the cabbage cultivar ECD11, were recently identified^29^. SNP markers linked to these genes/QTLs were developed. The presence of *Rcr4*, *Rcr8*, and *Rcr9* from *B. rapa* T19 and the QTLs, *Rcr_C03-1* and *Rcr_C08-1* from *B. oleracea* ECD11 in the newly resynthesized *B. napus* lines were confirmed using SNP markers genotyped with the KASP method (http://www.lgcgroup.com/), following the manufacturer’s instructions. PCR was performed in a StepOne Plus Real-Time PCR System (Applied Biosystem, Mississauga, ON).

### Statements

*B. rapa* breeding line, T19, was provided from the Saskatoon Research and Development Centre, Agriculture and Agri-Food Canada (SRDC, AAFC), in Saskatoon, Saskatchewan, Canada. The seeds of the resistant *B. rapa* line (T19), crossed with two *B. oleracea* lines, ECD11 and JL04, were provided by Dr. G. R. Dixon (The University of Warwick, Wellesbourne, Warwick, UK), and Dr. Zhen Huang (Northwest A&F University, Yangling, Shaanxi, China), respectively. Voucher specimen of these developed material from the current research has not been deposited yet in a publicly available herbarium. We have permission to collect the plants used in this study. All methods were performed in accordance with the Saskatoon Research and Development Centre, Agriculture and Agri-Food Canada (SRDC, AAFC), Saskatchewan, Canada guidelines/regulations/legislation

### Supplementary Information

Supplementary Tables.

Supplementary Figure S1.

Supplementary Figure S2.

Supplementary Legends.

## Data Availability

All data generated or analysed during this study are included in this published article [and its supplementary information files].
