# Supplementary figures and images for: Resynthesizing Brassica napus with race specific resistance genes and race non-specific QTLs to multiple races of Plasmodiophora brassicae
Source: Sci Rep. 2024 Jun 25;14:14627. doi: 10.1038/s41598-024-64795-x (PMC11199665; doi:10.1038/s41598-024-64795-x)

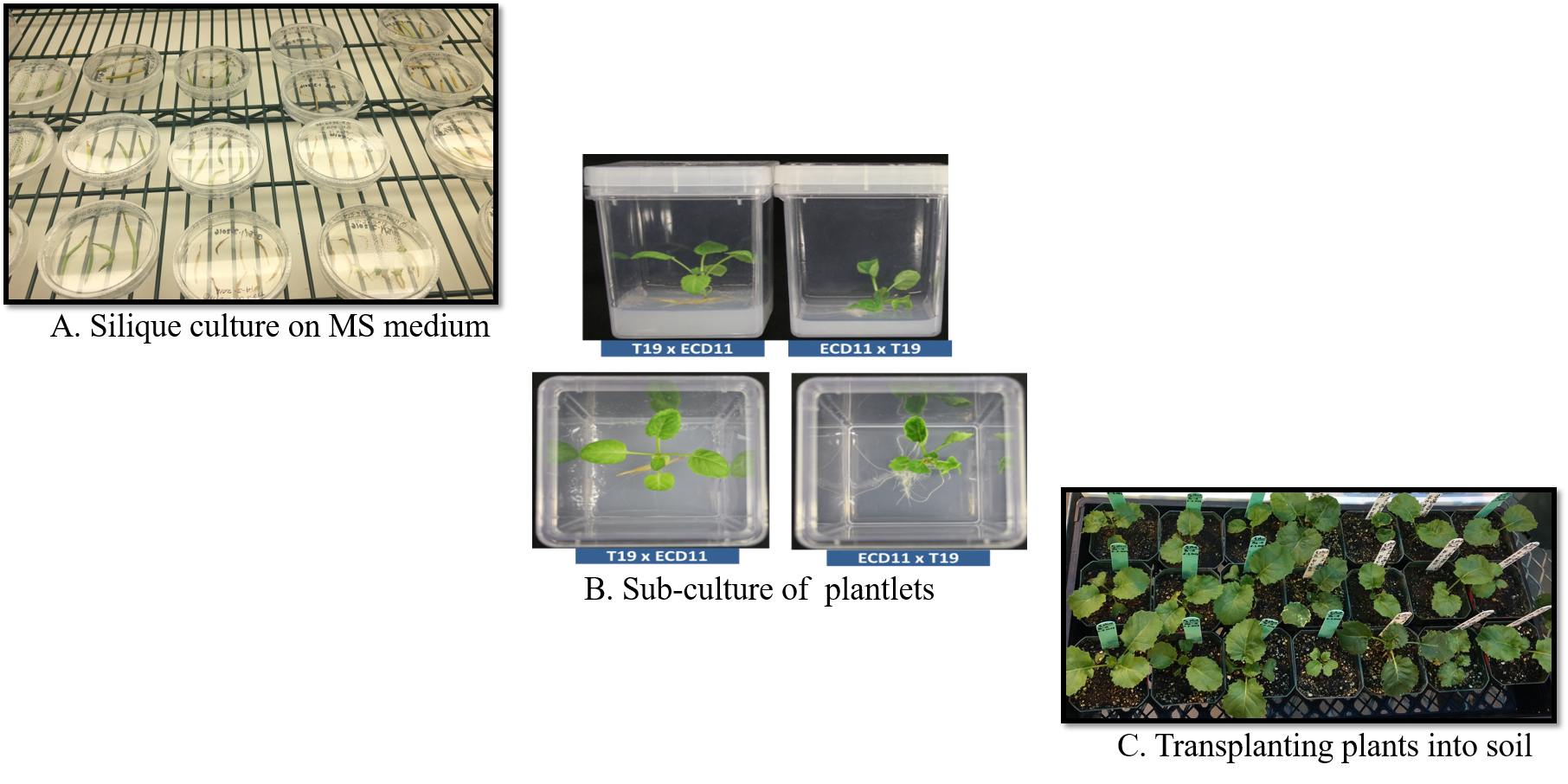

Supplement: Supplementary file 2 — Supplementary Figure S1. [file 41598_2024_64795_MOESM2_ESM.png]

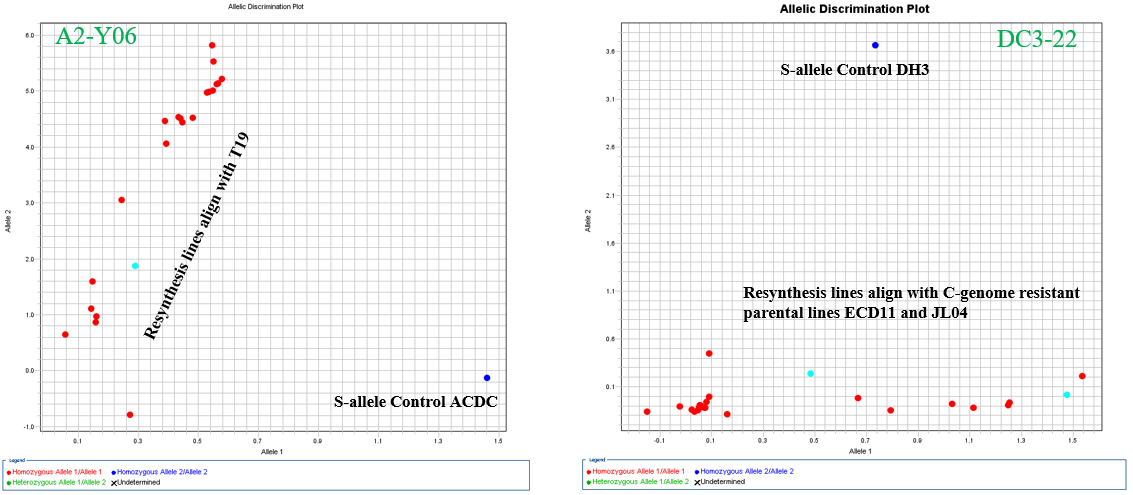

Supplement: Supplementary file 3 — Supplementary Figure S2. [file 41598_2024_64795_MOESM3_ESM.png]
