# Supplementary material for: Resynthesizing Brassica napus with race specific resistance genes and race non-specific QTLs to multiple races of Plasmodiophora brassicae
Source: Sci Rep. 2024 Jun 25;14:14627. doi: 10.1038/s41598-024-64795-x (PMC11199665; doi:10.1038/s41598-024-64795-x)
Supplement: Supplementary file 4 — Supplementary Legends. [file 41598_2024_64795_MOESM4_ESM.docx]

**Supplementary materials**

**Supplementary Table S1.** List and origin of strains, pathotypes and races of *Plasmodiophora brassicae* used in this study to test resynthesized and semi-resynthesized plants for resistance to clubroot.

**Supplementary Table S2.** Clubroot resistance of the resynthesized and semi-resynthesized *Brassica napus* lines to eight strains: 3A, 5G, 3H, 8J, 8P, PSI11, SK29 and 5X, representing 8 races of *Plasmodiophora brassicae*.

**Supplementary Table S3.** Scoring data for the KASP analysis: alleles from susceptible lines DH3 (*Brassica oleracea*) or ACDC (*Brassica rapa*) were recorded as “a” and from resistant parents (T19 or ECD11 or JL04) were recorded as “b”.

**Supplementary Figure S1.** Development of resynthesized *Brassica napus* (AACC) from interspecific hybridization between *Brassica rapa* (AA) parental line T19 and *Brassica oleracea* (CC) parental lines ECD11 and JL04. A) sterilized ovaries were placed on MS medium for embryo rescue; B) after 20 – 30 days, regenerating embryos were transferred to new MS agar medium in a plant tissue culture container for root and shoot development; C) plantlets with a height of 3 – 4 inches transplanted into four-inch pots containing Osmocote potting mix soil.

**Supplementary Figure S2.** Allelic discrimination plots of resynthesized *Brassica napus* (AACC) lines from the crosses T19 × ECD11 and T19 × JL04 with *Rcr8* linked SNP marker A2-Y06 and *Rcr_ECD11_C3* linked SNP marker DC3-22 were used for KASP analysis. ACDC and DH3 were used as susceptible control lines for the A- and C-genomes, respectively.
